# Lymphome plasmablastique de la muqueuse nasale isolée chez un patient immunocompétent en rémission complète après traitement multimodale: à propos d’un cas Africain et revue de la littérature

**DOI:** 10.11604/pamj.2020.37.22.21352

**Published:** 2020-09-05

**Authors:** Rajaa Tissir, Hanan Rais, Ilyas Tazi

**Affiliations:** 1 Service d’Hématologie, CHU Mohammed VI, Université Cadi Ayyad, Marrakech, Maroc,; 2 Service d’Anatomopathologie, CHU Mohammed VI, Université Cadi Ayyad, Marrakech, Maroc

**Keywords:** Lymphome plasmablastique, radiothérapie, nasal, VIH, Plasmablastic lymphoma, radiotherapy, nasal, HIV

## Abstract

Le lymphome plasmablastique est une variante agressive, récemment distincte du lymphome diffus à grandes cellules B. Initialement décrit dans la cavité buccale de patients immunodéprimés. Nous rapportons le premier cas d’un patient âgé de 54 ans qui a présenté 6 mois avant son admission un nodule de la cloison nasale saignant au contact et après éternuement. La tomodensitométrie (TDM) faciale avait montré un épaississement de la muqueuse nasale de 14mm d’épaisseur. La biopsie exérèse avait montré une prolifération tumorale faite de cellule d’allure plasmablastique avec à l’immunophénotypage: CD 138+, ki67 80%, EMA+, CD 79a+, CD 56+. Le bilan d’extension et la sérologie VIH était négatives. Vu la rareté de ce lymphome il n’y a pas de standards de traitement et la plupart des patients sont résistant au traitement avec un mauvais pronostic. Notre patient avait reçu 6 cures de chimiothérapie type CHOEP associé à une radiothérapie 40 gray en 20 fractions de 2 gray avec rémission complète ce qui est inhabituelle avec les cas décrits dans la littérature.

## Abstract (en)

Plasmablastic lymphoma is an aggressive variant of lymphomas recently distinct from diffuse large B cell lymphoma. It has been initially described as a disease affecting the oral cavity of immunocompromised patients. We here report the first case of a 54-year-old patient with nasal septum nodule, bleeding on contact and after sneezing which occurred 6 months before admission. Facial computed tomography (CT) scan showed thickening of the nasal mucosa of 14mm. Excisional biopsy showed tumor proliferation composed of plasmablastic cells with immunophenotypic features: CD 138+, ki67 80%, EMA+, CD79a+, CD 56+. Staging and HIV serology were negative. Given the rarity of this lymphoma there are no standard treatments and most patients have treatment-resistant lymphoma with poor prognosis. Our patient received 6 cycles of CHOP-like chemotherapy associated with 40 gy radiotherapy in 20 fractions of 2 gy with complete remission (unusual in the cases described in the literature).

## Introduction

Le lymphome plasmablastique (PBL) est une variante agressive, récemment distincte du lymphome diffus à grandes cellules B [1], initialement décrit dans la cavité buccale de patients immunodéprimés [2]. Ces dernières années plusieurs cas ont été rapportés impliquant diverses localisations chez des patients immunocompétents [2,3]. En raison de la rareté du lymphome plasmablastique, il n’y a pas de standard de traitement et la plupart des lymphomes sont résistant au traitement avec un mauvais pronostic. Nous rapportons le premier cas d’un lymphome plasmablastique de localisation nasale isolé chez un patient VIH négatif avec rémission complète sous traitement multimodal.

## Patient et observation

**Figure 1:**
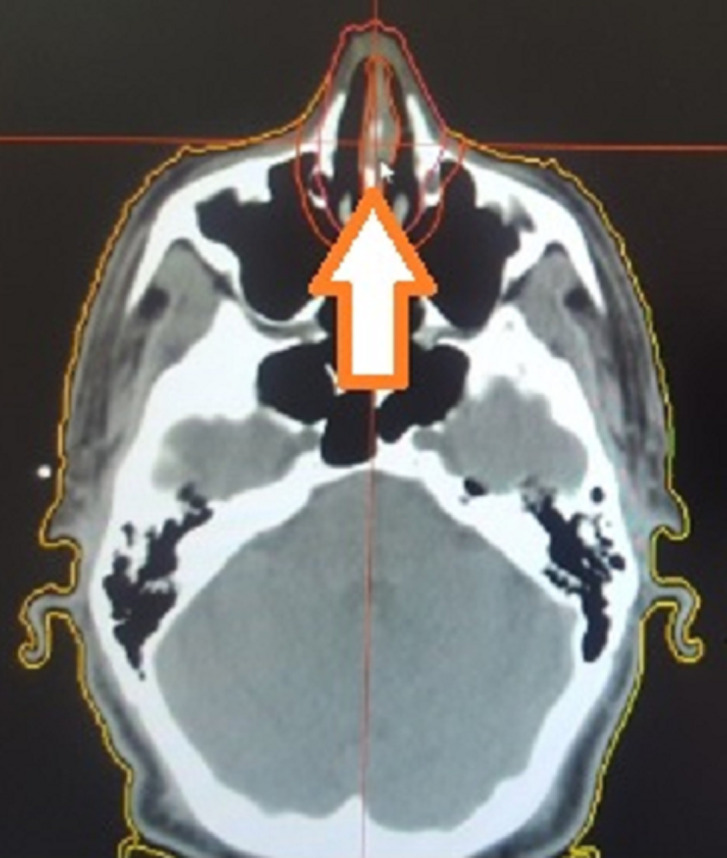
image scanographique montrant l’épaississement de la cloison nasal

**Figure 2:**
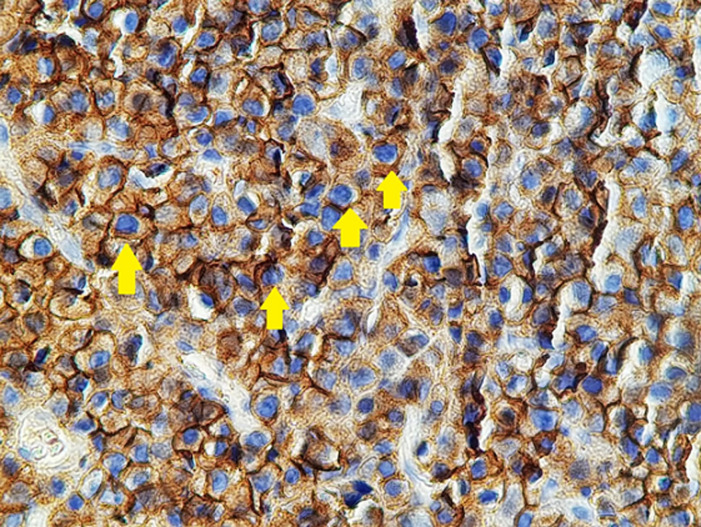
photo montrant une expression membranaire intense des cellules tumorales de l’anticorps CD138

**Figure 3:**
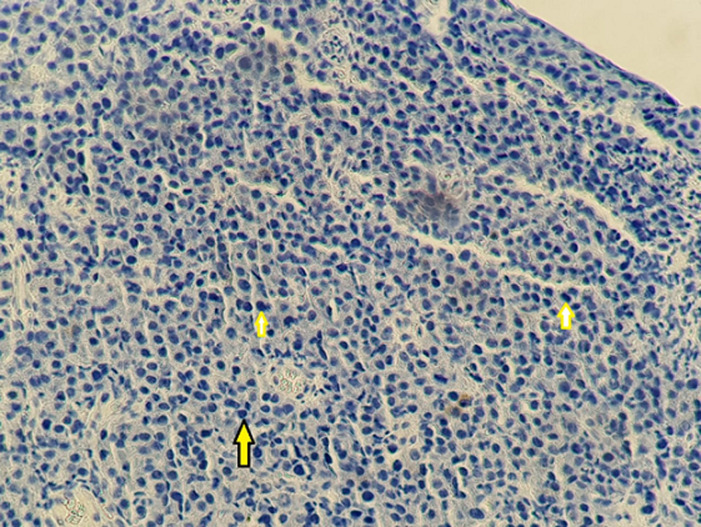
photo montrant une expression membranaire et focale des cellules tumorales de l’anticorps anti CD79a

**Figure 4:**
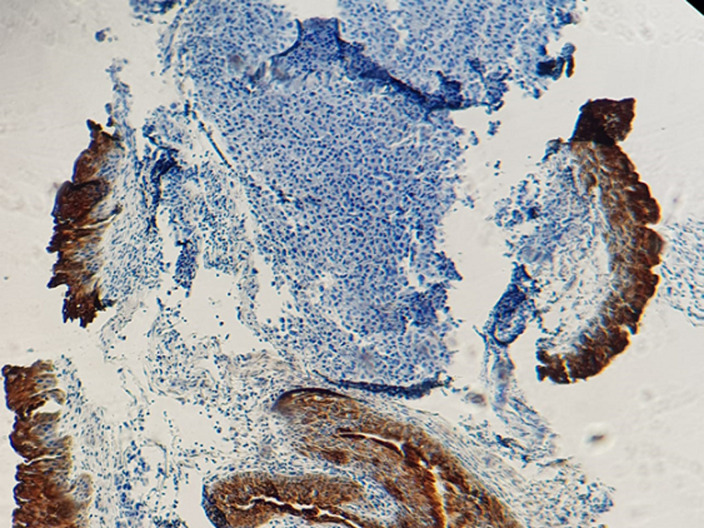
photo montrant l’absence d’expression de cellules tumorale de l’anticorps anti EMA

**Figure 5:**
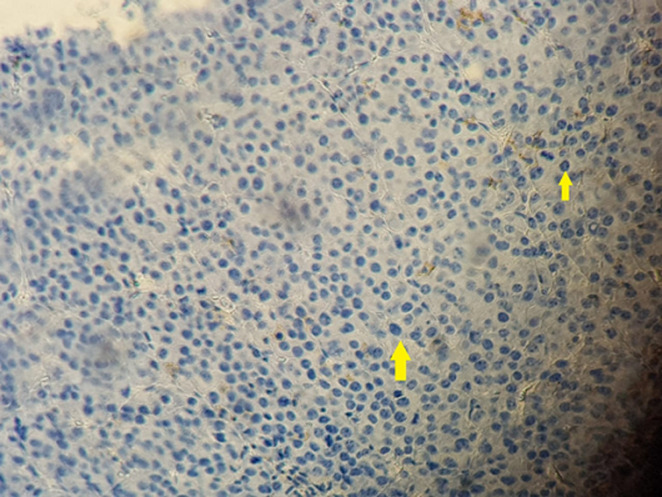
photo montrant une expression membranaire et focale des cellules tumorales de l’anticorps anti CD56

**Figure 6:**
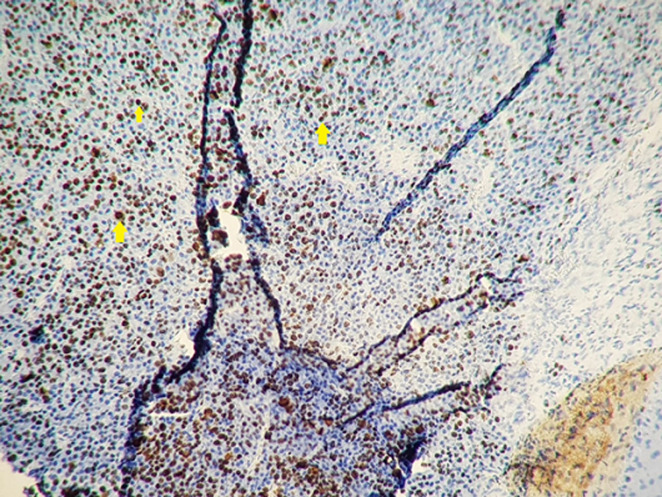
photo montrant une expression nucléaire intense de 80% des cellules tumorales de l’anticorps anti Ki67

Patient âgé de 54 ans qui a présenté 6 mois avant son admission un nodule de la cloison nasale de la narine droite saignant au contact et après éternuement dans un contexte de conservation de l’état général. La TDM faciale initiale avait montré un épaississement de la muqueuse de la cloison nasale de surface polyploïde de 14mm d’épaisseur (Figure 1). La biopsie exérèse avait montré une prolifération tumorale faite de cellule d’allure plasmablastique, CD138+, ki67 80%, EMA+, CD79a+, CD56+ (Figure 2, Figure 3, Figure 4, Figure 5 and Figure 6). Le bilan d’extension et la sérologie HIV étaient négatifs. Notre patient avait reçu 6 cures de chimiothérapie type CHOEP (cyclophosphamide, doxorubicine, vincristine, étoposide, prednisone) associé à une radiothérapie 40 gray en 20 fractions de 2 gray. L’évolution était marquée par une rémission complète de sa maladie.

## Discussion

Le PBL est un lymphome agressif rare décrit pour la première fois en 1997 par Delecluse *et al*. [4]. Il survient le plus souvent chez les sujets séropositifs avec un âge médian au diagnostic de 50 ans, notre patient était un homme de 54 ans. Initialement décrit dans la cavité buccale de patients immunodéprimés [5], plusieurs cas ont été rapportés ces dernières années impliquant diverses localisations extra ganglionnaires notamment muqueuses chez des patients immunocompétents [6], notre patient avait comme seule localisation la muqueuse nasale avec une sérologie HIV négative. Le diagnostic de PBL n’est pas facile vu les différents aspects morphologiques et immun phénotypiques posant des difficultés diagnostic avec d’autre lymphomes à différenciation plasmablastique [7,8]. Sur le plan histologique, ce lymphome se caractérise par la présence de cellules de grandes taille immature avec l’expression d’un ou plusieurs marqueurs associés au phénotype de cellules plasmatiques tels que CD138, CD38, BLIMP1, XBP-1 et IRF/MUM1 ainsi qu’une négativité de CD20 [7]. Vu la rareté du lymphome plasmablastique, il n’y a pas de standard de traitement et la plupart des lymphomes sont résistant au traitement avec un mauvais pronostic. Plusieurs schémas thérapeutiques ont été utilisés selon les cas publiés, incluant EPOCH, CODOX-M/IVAC et hyper-CVAD, vu que le CHOP n’est pas jugé efficace [9]. Notre patient avait reçu 6 cures CHOEP suivis d’une radiothérapie après décision de RCP avec rémission complète. La greffe de cellules souches hématopoïétiques est une option thérapeutique surtout chez les patients présentant une maladie récurrente ou réfractaire [10].

## Conclusion

Notre cas illustre une localisation inhabituelle du lymphome plasmablastique au niveau nasal isolé chez un patient VIH négatif avec rémission complète après traitement multimodale associant chimiothérapie et radiothérapie.

## References

[1] Elyamany G, Alzahrani AM, Aljuboury M, Mogadem N, Rehan N, Alsuhaibani O (2015). Clinicopathologic features of plasmablastic lymphoma: single-center series of 8 cases from Saudi Arabia. Diagn Pathol.

[2] Yasuhara R, Irié T, Shiozawa E, Yamochi T, Tanaka J, Kohno Y (2014). Plasmablastic lymphoma of the maxillary sinus with intraoral manifestation caused by direct alveolar bone infiltration in an HIV-negative patient. Pathol Int.

[3] Brahmania M, Sylwesterowic T, Leitch H (2011). Plasmablastic lymphoma in the ano-rectal junction presenting in an immunocompetent man: a case report. J Med Case Rep.

[4] Delecluse HJ, Anagnostopoulos I, Dallenbach F, Hummel M, Marafioti T, Schneider U (1997). Plasmablastic lymphomas of the oral cavity: a new entity associated with the human immunodeficiency virus infection. Blood.

[5] Arora N, Eule C, Gupta A, Li HC, Sadeghi N (2019). Clinicopathologic features, management and outcomes of plasmablastic lymphoma: a 10-year experience. Am J Hematol.

[6] Harmon CM, Smith LB (2016). Plasmablastic lymphoma: a review of clinicopathologic features and differential diagnosis. Arch Pathol Lab Med.

[7] Fernández-Álvarez R, Sancho JM, Ribera JM (2016). Plasmablastic lymphoma. Med Clin (Barc).

[8] Ahn JS, Okal R, Vos JA, Smolkin M, Kanate AS, Rosado FG (2017). Plasmablastic lymphoma versus plasmablastic myeloma: an ongoing diagnostic dilemma. J Clin Pathol.

[9] Kessler AJ, Marcellino BK, Niglio SA, Petersen BE, Malone AK (2019). A rare presentation of HIV-negative plasmablastic lymphoma: a diagnostic dilemma. Case Rep Hematol.

[10] Castillo JJ, Bibas M, Miranda RN (2015). The biology and treatment of plasmablastic lymphoma. Blood.

